# Deterioration of pre-existing hemiparesis due to injury of the ipsilateral anterior corticospinal tract

**DOI:** 10.1186/1471-2377-13-53

**Published:** 2013-05-29

**Authors:** Sung Ho Jang, Hyeok Gyu Kwon

**Affiliations:** 1 Department of Physical Medicine and Rehabilitation, College of Medicine, Yeungnam University, 317-1, Daemyung dong, Namku, Taegu 705-717, Republic of Korea

**Keywords:** Lateral corticospinal tract, Anterior corticospinal tract, Diffusion tensor imaging, Pontine infarct, Hemiparesis

## Abstract

**Background:**

The anterior corticospinal tract (CST) has been suggested as one of the ipsilateral motor pathways, which contribute to motor recovery following stroke. In this study, we report on a patient who showed deterioration of pre-existing hemiparesis due to an injury of the ipsilateral anterior CST following a pontine infarct, as evaluated by diffusion tensor tractography (DTT).

**Case presentation:**

A 55-year-old male patient showed quadriparesis after the onset of an infarct in the right pontine basis. He had history of an infarct in the left middle cerebral artery territory 7 years ago. Consequently, he showed right hemiparesis before onset of the right pontine infarct. Following this, his right hemiparesis deteriorated whereas his left hemiparesis newly developed. The DTTs for whole CST of the right hemisphere in the patient and both hemispheres in control subjects descended through the known CST pathway. By contrast, the DTT for the left whole CST of the patient showed a complete injury finding. The DTTs for the anterior CST of control subjects passed through the known pathway of the CST from cerebral cortex to medulla and terminated in the anterior funiculus of the upper cervical cord. However, the DTT for right anterior CST in the patient showed discontinuation below the right pontine infarct.

**Conclusion:**

It appeared that the deterioration of the pre-existing right hemiparesis was ascribed to an injury of the right anterior CST due to the right pontine infarct.

## Background

The corticospinal tract (CST) is the major neuronal pathway that mediates voluntary movements in the human brain [1,2]. The CST is generally divided into the crossed lateral CST and the uncrossed anterior CST. The anterior CST primarily innervates the musculature of the trunk and proximal extremities [1,2]. The anterior CST is considered to be one of the ipsilateral motor pathways from the unaffected motor cortex to the affected extremities, which contribute to motor recovery following stroke incidents [3].

Diffusion tensor tractography (DTT), which is derived from diffusion tensor imaging (DTI), allows for the visualization and investigation of neural tracts in three dimensions [4]. Since the introduction of DTI, DTT for the whole CST has been popular in the neuroscience field, but the identification of the anterior CST from whole CST has become possible recently [5-11].

In this study, we report on a patient who showed deterioration of pre-existing hemiparesis due to an injury of the ipsilateral anterior CST following a pontine infarct, as evaluated by DTT.

## Case presentation

### Ethics statement

All subjects provided signed, informed consent and our institutional review board approved the study protocol.

**Figure 1:**
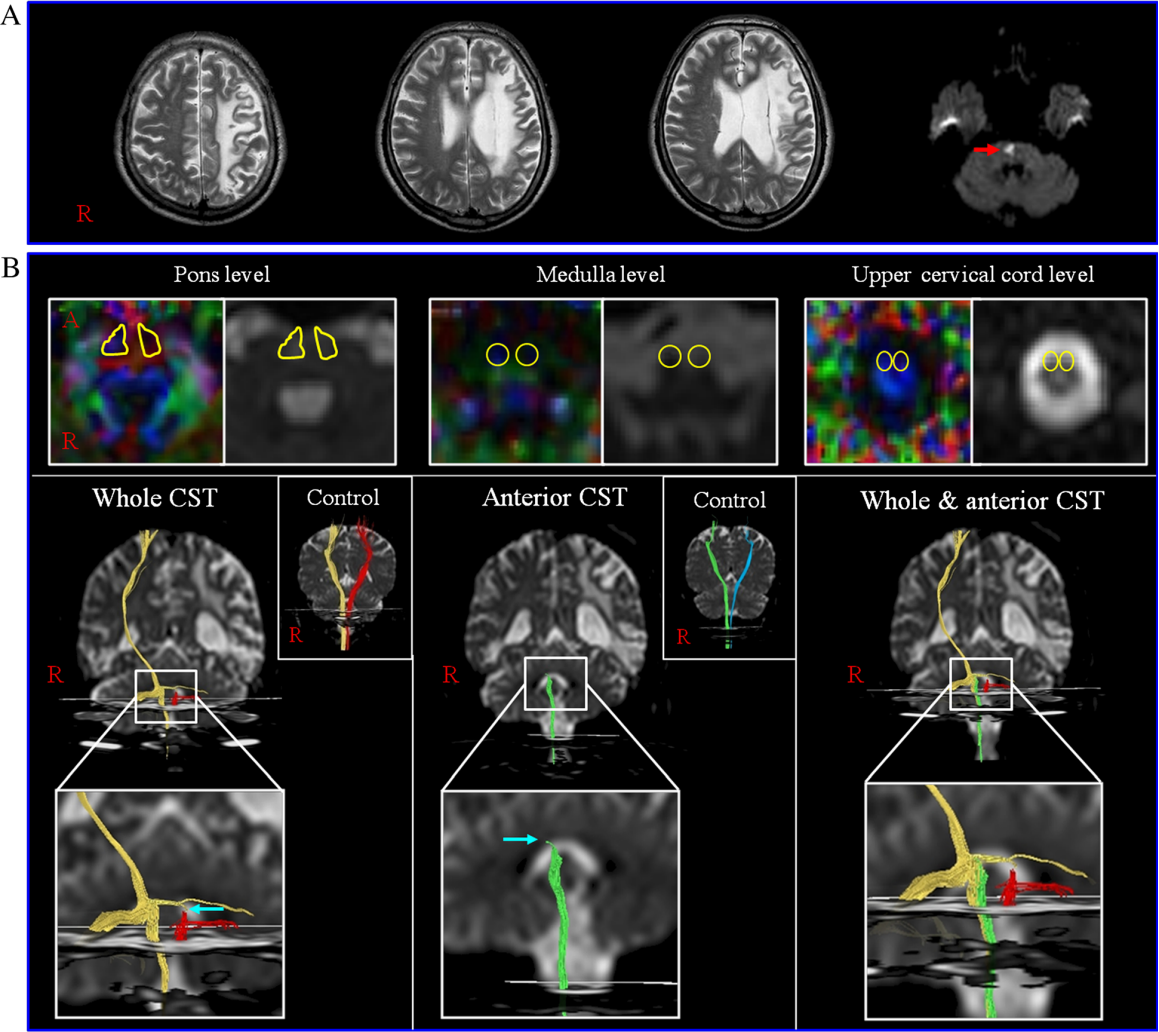
**Brain MRI and results of diffusion tensor tractography. A**) Brain MRI showing an old infarct in the left middle cerebral artery territory and a new infarct in the right pontine basis (arrow). **B**) Regions of interest for the whole and anterior corticospinal tract (CST)(yellow-lined circle) and results of diffusion tensor tractography. The whole CST and the anterior CST were constructed in the patient and a normal control subject (yellow: right whole CST, red: left whole CST, green: right anterior CST, blue: left anterior CST).

One patient and six right-handed sex-matched control subjects (6 male; mean age: 55.7 years, range: 51–58) with no history of neurologic disease participated in this study. A 55-year-old, right-handed male was consulted to the rehabilitation department of our university hospital for his quadriparesis. Motor function was evaluated using the Medical Research Council score (MRC) [12]: 0, no contraction; 1, palpable contraction but no visible movement; 2, movement without gravity; 3, movement against gravity; 4, movement against a resistance lower than the resistance overcome by the healthy side; 5, movement against a resistance equal to the maximum resistance overcome by the healthy side. The patient was diagnosed as having an infarct in the right pontine basis 3 weeks earlier at the neurology department of the same university hospital. He had history of an infarct in the left middle cerebral artery (MCA) territory 7 years ago. At that time, the motor function of his right extremities had recovered to the extent that he was able to walk with limping gait pattern independently and was able to move his right arm partially. After the onset of the right pontine infarct, his right extremities showed complete weakness (MRC; 0) although his left extremities showed partial weakness (MRC; 2–4). Brain MRI taken 3 weeks after the right pontine infarct showed an old infarct in the left MCA territory, involving the whole CST area at the corona radiata and a new infarct in the right pontine basis involving the CST area (Figure 1-A).

### Diffusion tensor tractography

A 6-channel head coil on a 1.5 T Philips Gyroscan Intera (Philips, Best, Netherlands) with single-shot echo-planar imaging was used for acquisition of DTI data. For each of the 32 non-collinear diffusion sensitizing gradients, we acquired 70 contiguous slices parallel to the anterior commissure-posterior commissure line. We scanned from the cortex to the middle of the second cervical vertebra body. Imaging parameters were as follows: acquisition matrix = 96 × 96; reconstructed to matrix = 192 × 192 matrix; field of view = 240 × 240 mm^2^; TR = 10,398 ms; TE = 72 ms; parallel imaging reduction factor (SENSE factor) = 2; EPI factor = 59; *b* = 1000 s/mm^2^; NEX = 1; and a slice thickness of 2.5 mm (acquired isotropic voxel size 2.5 × 2.5 × 2.5 mm^3^). Fiber tracking was performed using the FACT algorithm implemented within the DTI task card software [4,13]. The whole CSTs were determined by selection of fibers passing through two regions of interest (ROIs), which were placed on the CST area of the pons and upper medulla on the color maps [14,15]. By contrast, for reconstruction of the anterior CST, three ROIs were placed on the CST area of the pons and upper medulla (anterior blue color) and an additional ROI was placed on the anterior funiculus of the upper cervical cord on the color maps [16]. Termination criteria was fractional anisotropy (FA) < 0.2 and an angle change > 30°, as determined by a previous study on the optimal tractability threshold of FA [10,17].

The DTTs for whole CSTs of the right hemisphere in the patient and both hemispheres in the control subjects originated from the primary sensori-motor cortex and descended through the medullary pyramid along the known CST pathway. By contrast, the DTT for left whole CST of the patient showed a Wallerian degeneration to the left pons with discontinuation. The DTTs for anterior CSTs of both hemispheres in control subjects originated from the primary sensorimotor cortex and passed through the known pathway of the CST from the cerebral cortex to the medulla. They then terminated in the anterior funiculus of the upper cervical cord. However, the DTT for the right anterior CST of the patient showed discontinuation below the right pontine infarct (Figure 1-B).

## Discussion

In the current study, we evaluated the whole and anterior CSTs in a quadriparetic patient with a new right pontine infarct and an old left MCA infarct. According to our results, it appeared that the deterioration of the pre-existing right hemiparesis was ascribed to the injury of the right anterior CST following the new right pontine infarct, for the following reasons. First, the patient had been diagnosed with an infarct in the left MCA territory 7 years ago, involving whole CST in the left corona radiata [18]. Before the onset of the new right pontine infarct, the patient was able to walk independently and move his right arm partially. However, after the onset of the new infarct in the right pontine basis, the motor function of his right extremities deteriorated to complete weakness (MRC: 0), even though his left extremities showed partial weakness (MRC: 2–4). We presumed that some neural tract other than the left whole CST had been responsible for the partial motor function of the right extremities before the onset of new right pontine infarct. Considering that the new infarct was located in the CST area of the right pontine basis, the right anterior CST appeared to be the most plausible motor tract for deterioration of pre-existing right hemiparesis [14,19]. We confirmed the injury of the right anterior CST to be due to the pontine infarct by DTT.

Little is known about the ipsilateral motor pathway that causes deterioration of pre-existing hemiparesis in stroke patients [20]. In 2003, Ago et al. reported that a 59 year-old patient experienced deterioration of the already existing left hemiparesis that had resulted from a previous right putaminal hemorrhage due to a lacunar infarct in the left corona radiata [20]. They suggested that the new infarct in the left corona radiata caused damaged the uncrossed ipsilateral motor pathway, which had been responsible for the motor function of the left extremities, causing further deterioration of the pre-existing hemiparesis. However, they could not clarify the identity of the ipsilateral motor pathway of that patient.

## Conclusion

In conclusion, we showed a patient who showed deterioration of pre-existing hemiparesis by an injury of the ipsilateral anterior CST following a pontine infarct. As far as we know, this is the first DTT study to demonstrate ipsilateral motor weakness by an injury in the anterior CST of stroke patients. Several limitations of this study should be considered. First, DTI could lead to both false positive and negative throughout the white matter of brain because of complex fiber configurations [21]. Second, because it is a case report, the results of this study are limited to generalizations. Further studies to overcome these limitations should be encouraged.

## Consent

Written informed consent was obtained from the patient for publication of this case report and accompanying images. A copy of the written consent is available for review by the Editor-in-Chief of this journal.

## Abbreviations

CST: Corticospinal tract; DTI: Diffusion tensor imaging; DTT: Diffusion tensor tractography; FA: Fractional anisotropy; MCA: Middle cerebral artery; MRC: Medical research council; ROI: Region of interest.

## Competing interests

The authors declare that they have no competing interests.

## Authors’ contributions

SHJ: conceiving and designing the study, funding, data acquisition, manuscript development and manuscript writing. HGK: manuscript development, data acquisition, manuscript writing and manuscript authorization. Both authors read and approved the final manuscript.

## Pre-publication history

The pre-publication history for this paper can be accessed here:

http://www.biomedcentral.com/1471-2377/13/53/prepub
